# Welfare indicators for young dairy heifers managed with virtual fencing in northern Sweden

**DOI:** 10.3168/jdsc.2025-0993

**Published:** 2026-03-12

**Authors:** K. Liffen, G. Vaccargiu, K. Ren, J. Oliveira, M. Hetta, J.C.C. Chagas

**Affiliations:** 1 Department of Applied Animal Science and Welfare, Swedish University of Agricultural Sciences, 901 83 Umeå, Sweden; 2 Department of Animal and Veterinary Sciences, Aarhus University, 8000 Aarhus, Denmark; 3 Department of Applied Physics and Electronics, Umeå University, 901 87 Umeå, Sweden; 4 Department of Crop Production Ecology, Swedish University of Agriculture, 901 83 Umeå, Sweden

## Abstract

This study investigated the effects of virtual fencing (VF) on welfare indicators (i.e., BW, behavior, and hair cortisol) of young dairy heifers under northern Swedish conditions. Thirty heifers aged 126 to 294 d at the beginning of the experiment were allocated into 2 groups (n = 15): a control group managed with physical electric fencing, and a VF group. All heifers were fitted with VF collars before acclimatization on pasture. The 110-d experiment included a 5-d acclimatization period followed by three 35-d grazing periods conducted in different paddock areas (full paddock, one side, and the opposite side), each comprising a 6-d learning phase and a 29-d post-learning phase. Collar data for the VF group were analyzed for each learning phase and subsequent days in each period. Animal behavior was evaluated as scan sampling observations. Herbage samples were collected at the start and end of each period to evaluate herbage mass and chemical composition for potential pasture utilization assessment for both groups. The heifers were weighed at the beginning and at the end of the trial, and tail hair samples were collected at the end of the experiment for cortisol analysis. Over the course of the experiment the number of recorded audio cues increased and electrical pulses decreased. No significant differences were observed in behaviors such as grazing or lying down ruminating between the VF and control groups, nor were there significant differences in herbage mass or composition. Body weight gain was lower and hair cortisol concentrations were significantly higher for heifers managed with VF. Virtual fencing collars maintained reliable functionality throughout the 110-d trial under summer grazing conditions, and heifers aged 6 to 9 mo rapidly learned and adapted to the system. Although slightly higher cortisol concentrations and differences in growth were observed in the VF group, no behavioral evidence of impaired welfare was detected. Overall, VF demonstrated functional reliability under practical conditions; however, further research is needed to clarify the physiological and welfare implications of VF use in young heifers.

## Highlights

• Virtual fencing technology maintained its function under northern Swedish summer conditions.
• Audio cues increased, and electrical pulses decreased, showing effective learning.
• No group differences were found in grazing or lying ruminating behaviors.
• Potential effects on young heifer physiological responses.
• Unclear evidence of impaired animal welfare under virtual fencing use.

## Graphical Abstract

Virtual fencing may offer a cost-effective alternative for grazing systems; however, several aspects remain understudied, including its reliability under varying environmental conditions and its suitability for young cattle. This study evaluated virtual fencing in dairy heifers aged 6 to 9 months over a 110-day experiment divided into 3 periods associated with paddock changes. Heifers were assigned to either a control group managed with electric fences or a virtual fencing group. Throughout the experiment, collars maintained charge and functionality under northern Swedish summer conditions. Audio cues increased, and electrical pulses decreased, indicating that the heifers learned the virtual boundary and that the system effectively contained them. Heifers managed with virtual fencing showed reduced weight gain and higher hair cortisol concentrations compared with control, but behavior was not affected. These findings demonstrate the potential of virtual fencing while highlighting the need for further research on its long-term effects on young cattle. CNT = control group; VF = virtual fencing

**Figure:**
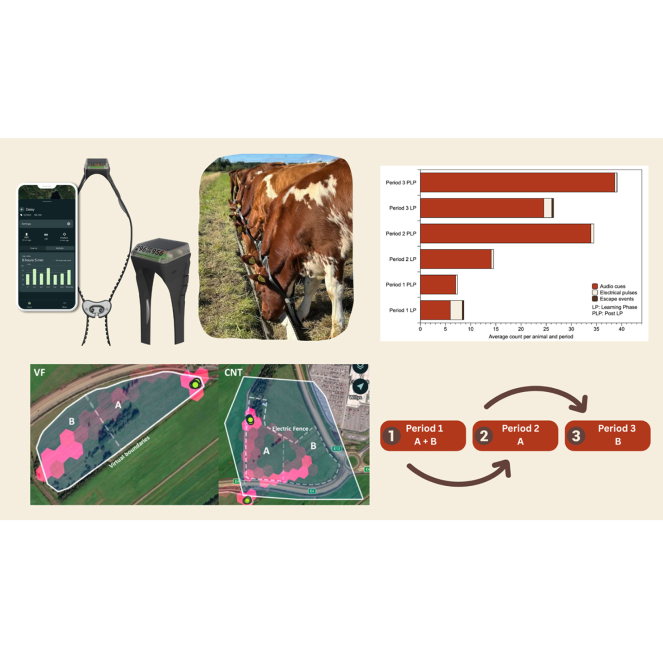

In high-latitude regions such as northern Sweden, the combination of mild summer temperatures and extended daylight offers favorable grazing conditions for cattle. Consumers also view pasture-based grazing systems as more aligned with cattle natural behaviors and therefore associated with positive animal welfare (Mee and Boyle, 2020). Although producer and consumer interests in grazing and pasture use are increasing, the costs of monitoring animals and maintaining fencing remain major challenges for competitive pasture-based livestock production systems in Sweden (Karlsson et al., 2024). In this context, precision livestock farming tools such as virtual fencing (**VF**) may offer solutions by reducing labor costs and improving management practices. Virtual fencing technology uses neck collars equipped with global navigation satellite systems (**GNSS**) and telecommunication functions, where audio cues (**AC**) and electrical pulses (**EP**) replace traditional electric fencing.

Despite increasing research on VF, some aspects remain understudied, including VF reliability under differing environmental conditions (Fuchs et al., 2025). Subarctic areas often experience cloudy weather and relatively low solar radiation during mild summers (Köppen, 1936; Peel et al., 2007), which may affect connectivity and reduce the functionality of solar-powered collar batteries. Another research gap is the suggested animal age limitation for using the collars, as little is known about VF effects on young cattle. Recent studies have found no long-term welfare impairment when VF is used (Hamidi et al., 2022; Fuchs et al., 2025), but most studies involve cattle above 11 mo of age. To address these research gaps, we aimed to investigate the effects of VF using welfare indicators (i.e., BW, behavior, and hair cortisol) on young dairy heifers under northern Swedish conditions. We hypothesized that young heifers would learn and adapt to VF without animal welfare impairment.

The study involved 30 Nordic Red dairy heifers aged 126 to 294 d at the beginning of the experiment (222 ± 55.7 kg BW; mean ± SEM) and was conducted from May to September 2025 at Röbäcksdalen experimental farm (63°45′ N, 20°17′ E), which is part of the Swedish Infrastructure for Ecosystem Science within the Swedish University of Agricultural Sciences. Air temperature, relative humidity, precipitation, and radiation were monitored throughout the trial using meteorological data from Röbäcksdalen Research Station (SITES, 2025).

Before the experiment, all heifers were fitted with VF neck collars weighing ~950 g (Monil AS, v2.0, 2024; GNSS-equipped neck collars with solar-powered battery). The collars delivered EP at 2,000 V and 0.25 J for 0.1 s. The Monil VF system provides an AC after the boundary is crossed. If the AC is ignored and the animal does not return within the VF boundary, an EP is administered. A maximum of 3 AC and 3 EP are delivered before the animal is classified as escaped.

Heifers were randomly assigned by the researchers into 2 balanced groups (n = 15 per group) based on age and BW: a control group managed with physical electric fencing and a VF group contained with virtual boundaries on all sides. The 2 groups were kept in separate paddocks for 110 d, each enclosed by physical fencing with stand-off wires to ensure animal safety near the adjoining highway. Throughout the trial all heifers had natural shade available and ad libitum access to water and mineral supplementation (Lantmännen, Nötfoder Mineralfoder Effekt Intensiv). Both groups were also provided with 20 kg/d of concentrate feed (Lantmännen, Idol).

The 110-d experiment began with a 5-d acclimatation period, followed by three 35-d experimental grazing periods, each consisting of a 6-d learning phase (**LP**) and a 29-d post-learning phase (**PLP**). In the VF group, virtual boundaries, AC, and EP were activated on d 1 (start of LP 1), whereas collars in the control group operated in the same mode but with the VF boundary drawn outside the paddock so heifers could not interact with the boundary (Figure 1). This was done to ensure that the accuracy of the GNSS position to track the collars was identical for both groups. For experimental period 1, both groups had access to the entire paddock (6.8 ha each), where, for LP 1, the VF was novel to the heifers. For periods 2 and 3, each paddock was divided into 2 equal sections (paddocks A and B; 3.4 ha each), where the VF boundaries were moved to divide the paddock into 2 different halves for LP 2 and LP 3. Thus, in period 2, the heifers grazed paddock A, and in period 3 paddock B. This setup (Figure 1) supported practical pasture management and herbage allowance while enabling assessment of whether heifers underwent an LP each time the VF boundaries were moved. Over the course of the study, 4 heifers were removed from the experiment: 2 heifers from the VF group for receiving more EP than allowed in a day (≥5), in accordance with our ethical permit; and 2 heifers from the control group, to keep the groups equally balanced with the same stocking rate per paddock.

**Figure 1:**
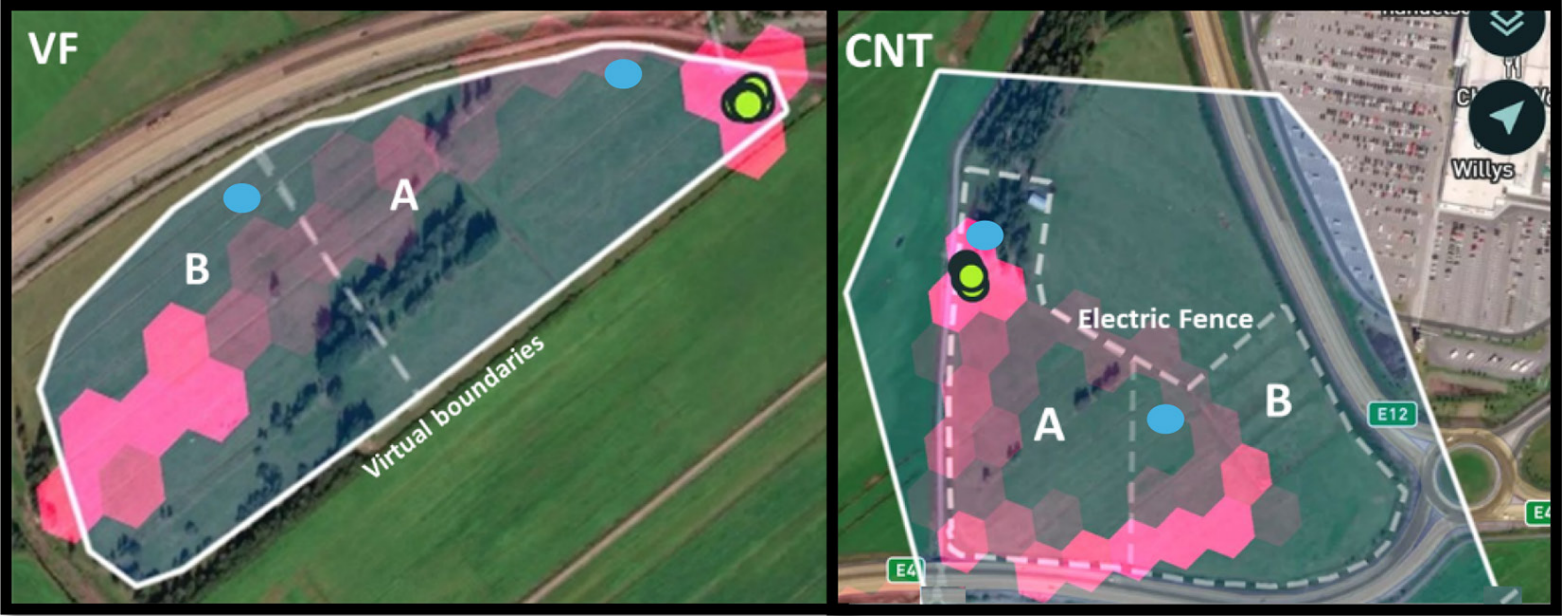
Map view from the Monil app user interface for each experimental field: VF (virtual fencing group) and CNT (control group). The layout shows the total paddock used in period 1 and paddocks A (period 2) and B (period 3). On the VF map, the white dashed lines indicate the virtual boundary divisions applied during periods 2 and 3. On the CNT map, the white dashed lines indicate the total electric-fenced paddock and its subdivision during periods 2 and 3. Blue circles represent water sources, green dots indicate global positioning system (GNSS) collars (individual animals), and pink shading illustrates the heatmap (GNSS position density over time) for the specific day the screenshot was taken from the Monil app, for illustrative purposes only.

The VF collar data were recorded and stored on the Monil server for each animal over the length of the experiment, including battery charge, animal position, events (AC, EP, escape events), and EP status (delivered or not delivered). The total numbers of AC, EP, and escape events were summarized for each experimental period as LP and PLP. The data were analyzed in SAS (version 9.4, SAS Institute Inc.) using PROC SQL and are presented descriptively. Scan sampling for behavioral observations was carried out every 10 min over an 8-h period on 5 nonconsecutive days (d 27–28 in periods 1 and 2 PLP, and only d 28 in period 3 PLP due to weather conditions). Two trained observers alternated between paddocks across observation days to avoid observer bias (interobserver reliability; intraclass correlation coefficient = 0.904). An ethogram containing 19 behaviors was used, and all behaviors were recorded on a group level. The 4 most frequently observed behaviors (grazing, lying down ruminating, lying down, and lying down curled up) were analyzed using PROC GLM, with observation days as replicates (n = 5) and experimental groups as fixed effects in the model.

Pasture DM content and herbage mass (kg DM/ha) were assessed using the quadrat method combined with ruler sward-height measurements. Twelve sample points were randomly selected in each paddock for each sampling occasion, with sampling conducted at the beginning and at the end of every experimental period according to weather conditions (n = 2 per period per group). Herbage samples were dried for 48 h at 60°C in a forced-air oven and then milled through a 1-mm sieve (Wiley mill). The wet and dried weights were used for calculating pasture DM and herbage mass, and milled samples were analyzed for CP, OM, NDF, indigestible NDF, and ME (Eurofins AB, Östersund, Sweden). Herbage mass and chemical components were statically analyzed using PROC GLM in SAS, considering experimental groups, period, and the group × period interaction as fixed effects in the model.

Body weight was measured with an electronic livestock scale at the beginning and end of the trial (Dini Argeo). Initial BW, BW gain (**BWG**), final BW, and ADG were analyzed with PROC GLM, including experimental groups as fixed effect in the model. Initial BW was tested in the model as a covariate and was excluded whenever it did not demonstrate statistical significance.

At the end of the trial, hair samples from the tail were collected for cortisol analysis following the clipping method for “location E” from Moya et al. (2013). The hair cortisol analysis followed Tamminen et al. (2021), with modifications to the buffer volume (150 μL) and the methanol mixing time (21 h). Once samples had been processed, the data were analyzed by removing outliers (Grubbs test; n = 2 removed, leaving a total of n = 12 per group). Cortisol data (pg/mg) were not normally distributed, so a Wilcoxon rank-sum test was performed.

Throughout the trial, all VF collar connectivity and battery levels were monitored daily via the VF application installed on both a smartphone and a tablet. On one occasion, a collar from the VF group lost connection for several hours; however, this did not affect overall monitoring. Before the start of the experiment, all collars were fully charged, displaying 100% battery in the applications. None of the collars showed a reduction in battery level from 100%, despite the mild air temperatures (8.32 to 22.4°C) and the medium-low incoming shortwave radiation recorded during the 110-d trial (averaging 201.7 W/m^2^), with 50 d below 200 W/m^2^ and 20 of those days below 100 W/m^2^.

Audio cues increased throughout the experiment in both the LP and the PLP (Figure 2). The LP in periods 1, 2, and 3 showed average AC counts per animal and period of 5.95 ± 5.443, 14.1 ± 5.72, and 24.5 ± 5.56 (mean ± SEM), respectively. The highest average AC was observed in PLP period 3 (32.0 ± 2.97). Instances of EP decreased over time, although a slight increase occurred between periods 2 and 3. Current literature on VF has included young cattle within broader experimental designs; however, relatively few studies have specifically focused on early-postweaning heifers in the narrower age range of 6 to 9 mo. Moreover, findings remain inconsistent regarding the effects of VF on behavior, physiology, and growth. The VF collar responses observed in the present study align with those reported by Verdon et al. (2020), who found that young heifers (~100 d at trial start) exhibiting more frequent fence interactions (AC) received fewer EP over time. This learning-related shift explains the increase in AC observed throughout our study, particularly following boundary adjustments. The heifers explored the new area but had already associated AC with EP and therefore avoided receiving EP, demonstrating that they had successfully learned the VF boundaries.

**Figure 2:**
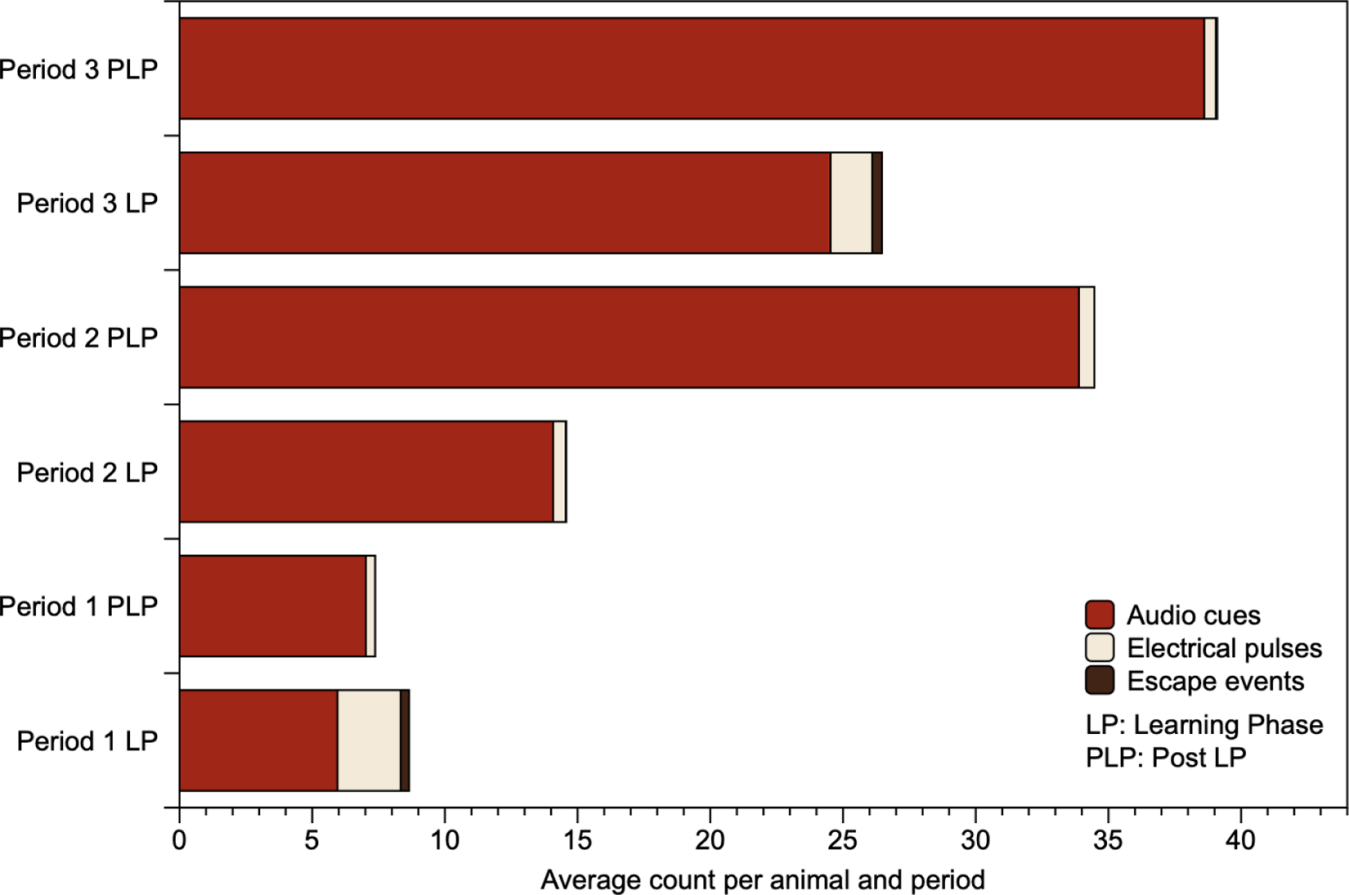
Average count per animal and period of collar audio cues, electrical pulses, and escape events over the course of the study, divided into learning phases (LP) and post-learning phase (PLP) periods.

It was noted during the trial that 2 heifers repeatedly tested the VF boundaries. This behavior appears to be a common occurrence, where certain individuals are willing to continue crossing VF boundaries (Campbell et al., 2020). Initially, one heifer exhibited this pattern in period 2. Following her removal due to high EP, another heifer developed a similar pattern in period 3. These heifers exceeded the group average in warnings, showing ~10 to 15 consecutive days in periods 2 and 3 with AC daily counts above 200. Their maximum daily AC reached 511 and 619. As a result, both heifers also received more EP than the group average, leading to their removal from the experiment.

Another observation from the VF collar data was the occurrence of mass escape events in period 3, when the heifers were grazing in paddock B (Figure 1). Mass escape events consisted of all heifers escaping the VF boundary and spending several hours outside the VF before returning on their own to within the VF boundaries. One event occurred during the LP and 2 during the PLP. Paddock B had a back corner hidden by a ditch and trees and was more prone to hosting wild animals. Thus, we suspect that the heifers may have been startled by wild animals or domestic dogs passing near the field, which is common around the research farm. However, the actual motivation for the mass escape events could not be identified. Further work is required on individual animal fence interactions and mass escape behavior to fully understand individual animal learning and to identify ways of adapting the technology to herd management and farm dynamics.

We found no significant differences in behaviors between VF and control groups (*P* > 0.05), where grazing (VF 39.4% ± 3.17 SEM; control 34.9% ± 3.17 SEM) and lying down ruminating (VF 26.2% ± 1.96 SEM; control 21.9% ± 1.96 SEM) were the most observed behaviors for both groups, presented as frequency (%) of total behavioral expression. Hamidi et al. (2022) observed no behavioral effects of VF management in heifers aged 14 to 16 mo, whereas Campbell et al. (2019) reported reduced lying time in cattle managed with VF compared with those under electric fencing. The behavioral results in our study were not significant, which may suggest that VF did not have a negative effect on animal behavior. This is particularly relevant because lying behaviors are directly associated with welfare in cattle (Wagner et al., 2018), and thus behavior was not adversely affected in this study.

We aimed to determine whether herbage use or grazing differed between groups; however, no such differences were detected with the applied methods. Paddock herbage mass and chemical composition did not differ between groups, and no group × experimental period interaction was observed (*P* ≥ 0.230). As expected, experimental periods differed significantly (*P* < 0.001), reflecting the different grazing settings implemented throughout the study (Figure 1).

Initial BW did not differ between groups (*P* = 0.135), confirming that the experiment began with balanced groups. However, final BW, BWG, and ADG were significantly lower in the VF group (*P* < 0.001; Table 1). The literature reports inconsistent findings. Hamidi et al. (2022) observed no effect of VF on BWG in heifers aged 14 to 16 mo, and Verdon et al. (2021) similarly reported no effect of VF on growth performance in a case study. In contrast, Campbell et al. (2019), consistent with the present study, found lower BWG in Angus steers aged 12 to 14 mo managed with VF (mean ± SEM: VF 32.38 ± 2.20 kg vs. electric tape 48.94 ± 2.45 kg). However, this effect was observed in only 1 of the 2 cohorts and may have been influenced by differences in pasture allowance and feed intake.

**Table 1:** Body weight parameters (kg) at the start (BWi) and end of the trial (BWf) for control and virtual fencing groups, with changes in BW expressed in total gain (BWG) and ADG over the trial

Parameter	Control	Virtual fencing	SEM	*P*-value
BWi	192	221	13.2	0.135
BWf^1^	312	284	6.14	<0.001
BWG	111	85.3	5.56	<0.001
ADG	1.00	0.76	0.05	<0.001

1 Covariate kept in the model as significant.

Hair cortisol concentrations were significantly higher (*P* = 0.036) for the VF group (18.6 ± 2.12 pg/mg; mean ± SEM) compared with the control group (11.6 pg/mg ± 2.12). This is in direct contrast to Confessore et al. (2024), who found that VF interactions did not significantly affect hair cortisol concentration for younger first-lactation cows (first day: 0.06 ± 0.05 pg/mg; last day: 0.07 ± 0.01 pg/mg). Although, in general, young cattle have higher concentrations of hair cortisol (15-d-old heifers: 114.5 6 14.43 pg/mg) when compared with older cattle (2-yr-old cows: 12.15 6 1.85 pg/mg; González-de-la-Vara et al., 2011), and hair color significantly affects cortisol concentration, which was not considered during this trial (Shi et al., 2021). It is important to highlight that cortisol as a stress marker does not always indicate poor welfare, as stress is not necessarily negative and can also be positive (Koolhaas et al., 2011). Moreover, many factors unrelated to stress can increase cortisol levels (e.g., exercise; Krumrych et al., 2018). Overall, our findings indicate that younger heifers managed with VF collars exhibited slightly higher cortisol concentrations. Whether this reflects increased excitement, physical activity, or a transient stress response requires further investigation on VF long-term effects in young cattle.

The differences in BW results suggest that the heifers in this study managed with VF may have diverted energy away from maintenance and growth requirements, possibly due to a shift in grazing behavior and an increase in exercise (movement; Di Marco and Aello, 2001), which could help explain the higher cortisol values observed (Krumrych et al., 2018). The animals may have spent more time roaming and less time effectively eating during grazing to search for their preferred herbage while avoiding EP through the association of AC (Ksiksi and Laca, 2002). However, this could not be fully evaluated in this study, so this explanation remains an inference, and the results should be interpreted with caution because physiological stress responses are complex and multifactorial. Thus, the findings suggest a potential physiological response without clear evidence of impaired animal welfare. Therefore, longer-term studies are warranted to determine whether VF alters cattle grazing behavior over time and to further evaluate whether hair cortisol is an appropriate noninvasive indicator of welfare in this context. In cattle, fecal cortisol metabolites are more commonly used as a welfare indicator (Campbell et al., 2019; Hamidi et al., 2022), and comparative evaluation of these measures would strengthen interpretation of physiological responses to VF.

Nevertheless, it is important to note that during the last 2 wk of period 2 (PLP), the electric fence in the control group experienced a power outage. As a result, the heifers crossed into paddock B, where fresh herbage was available following mowing. Although paddock B had also been mowed for the VF group, their collars functioned as intended and prevented access to the adjacent area. The differences observed in BWG, final BW, and ADG between groups (Table 1) may therefore be partially explained by compensatory gains in the control group following access to the newly available herbage during the fence failure. Such events are not uncommon under practical farming conditions and illustrate the management challenges associated with physical fencing, as well as the potential operational advantages of VF systems.

In conclusion, the VF collars maintained sufficient charge and functionality throughout the 110-d trial under local summer climate conditions, and heifers aged 6 to 9 mo rapidly learned to respond to VF warnings. Although slightly higher cortisol concentrations were observed in the VF group, this suggests a physiological response without clear evidence of impaired animal welfare. Given the moderate sample size adopted and the occurrence of fence failure during the study, BW responses should be interpreted with caution. Overall, under practical grazing conditions, VF demonstrated functional reliability and practical applicability in young heifers.
